# How to Construct an Upper Vulcanite Denture Quickly

**Published:** 1902-11-15

**Authors:** C. H. Warboys


### HOW TO CONSTRUCT AN UPPER VULCANITE DENTURE QUICKLY.

BY C. H. WARBOYS, D.D.S.

Read before the Southwestern Michigan Dental Society, September 9, 1902.

To know how to construct a set of teeth in a very short time, is a very convenient thing sometimes, and the object of this paper is to give you a plan by which it may be accomplished, and at the same time do so in such a way that you will be able to proceed with great exactness. This description will be for a full upper denture.

A tray is selected that is smooth, and fits the jaw as closely as it is easy to use on the case, and then an impression is taken in modeling compound. This impression should be thoroughly chilled and then carefully slipped from the tray; with a sharp knife now trim the impression to just the outline that the plate is to have, and do any relieving along the hard ridge that may be considered necessary; at the edges and across the back, make it quite thin; now try it in the mouth and if it is all right, remove an cover the ridge with soft wax and replace in the mouth to get the bite and contour.

At this point I wish to call your attention to two anatomical points of the human skull, that we seem to forget exists when we are constructing an artificial denture for the upper jaw, namely, the canine ridge and canine fossa. These should be reproduced, and one is just as important as the other in order to restore he face to its former appearance.

Another point is the correct distance between the jaws. This is difficult to ascertain, where there is no record of measurements. It is my practice to take the distance between the nose and the point of the chin with a pair of dividers, and from the same point under the nose to the cutting edge of a central upper incisor, with the color, width and length of the tooth, all of which are registered with the patients name before the extracting is done. These measurements are valuable when the time comes for constructing the plate. Having secured the articulation from the bite, the incisors are now waxed to position on the impression, and arranged as desired; the bicuspids and molars are set and articulated to the lower teeth on one side first, and should be firmly waxed, then those of the other side are set up and articulated; all this being tried in the mouth from time to time. Build up the gum contour in wax and with a sharp scraper cut the modeling compound as thin as you wish the plate, and it is now ready for investment in the flask, which I open or separate with warm water to heat the compound, and continue as usual with a rubber plate.

